# Meetings of Societies

**Published:** 1909-12

**Authors:** 


### MEETINGS OF SOCIETIES.

### Bristol Medico-Chirurgical Society.

*Annual Meeting, October 13th*, 1909.

After a vote of thanks to the retiring President, Dr. Michell Clarke, had been passed, Dr. James Swain took the chair and gave an introductory address (see p. 289). Later a cordial vote of thanks was given to Dr. Swain for his able and interesting address.

The Honorary Secretary and Editorial Secretary, Dr. J. A. Nixon, read the following annual report :—

There is a balance on hand on the general account of £81 14s. 8d. From members' subscriptions the receipts were £173 5s. Balance from previous year, £94 14s. 5d. Interest on deposit account, £3 0s. 2d. The expenses have amounted to £189 10s. 5d., of which the chief items are: Library Grant, £65; Rent, etc., £57 6s. Payment for members to *Journal*, £48 10s.

The balance of the *Journal* Account standing to the credit of the Society last October was paid to the Editorial Secretary to meet a deficit on the *Journal*, and there are no funds in this account at present.

During the year 1908–9 thirty new members joined the Society, twenty-two resigned, ten died. On October 1st, 1909, there were 166 members.

The meetings have been well attended, the average, excluding visitors, being forty-one.

The Hon. Librarian, Mr. C. K. Rudge, read the following report:—

The Library of the Bristol Medico-Chirurgical Society now contains, including duplicates, 11,098 volumes. 302 volumes have been added during the year, and 206 duplicate volumes have been sold. The Library is in regular receipt of 188 periodicals.

Under the new regulations permitting members to take out books other than duplicates 832 issues of books have been loaned to ninety borrowers during the year. This number includes thirty-six volumes borrowed from Lewis's Library. The total amount received in fines was 12s. from sixteen borrowers.

The Bristol Medical Library, to which members of the Society have access, now consists of 21,235 volumes, and it is in receipt of 251 current periodicals. These are contributed by the constituent libraries in the following manner:—

BOOKS.

| | |
|---|---|
| Bristol Medico-Chirurgical Society .. | 11,098 |
| Faculty of Medicine, University .. | 8,374 |
| Bristol Royal Infirmary .. .. .. | 1,189 |
| Bristol General Hospital .. .. .. | 574 |
| | 21,235 |

PERIODICALS.

| | |
|---|---|
| Medico-Chirurgical Society .. .. .. | 194 |
| Faculty of Medicine .. .. .. .. .. | 57 |
| | 251 |

During the year 382 volumes have been added to the Library.

The following are the officers for the Session 1909–10: President—James Swain, M.D., M.S.Lond. President-elect—B. M. H. Rogers, M.A., M.D.Oxon. Committee (*Ex officio*)—J. Michell Clarke, M.A., M.D.Cantab. (retiring President); R. Shingleton Smith, M.D.Lond. (Editor of *Journal*); C. K. Rudge, M.R.C.S. (Hon. Librarian); J. A. Nixon, B.A., M.B.Cantab.

(Editorial Secretary). (*Elected*)—J. P. Bush, C.M.G. (Ex-President) ; J. Taylor, M.R.C.S. (Ex-President) ; P. Watson Williams, M.D.Lond. ; G. Parker, M.A., M.D.Cantab. ; I. Walker Hall, M.D.Vict. ; F. Lace, F.R.C.S. ; J. Lacy Firth, M.D., M.S. Lond. ; C. A. Morton, F.R.C.S. Hon. Secretary—J. A. Nixon, B.A., M.B.Cantab.

---

**RECEIPT AND EXPENDITURE ACCOUNT OF THE BRISTOL MEDICO-CHIRURGICAL SOCIETY, SESSION 1908-9.**

| | £ | s. | d. | | £ | s. | d. |
|---|---|---|---|---|---|---|---|
| Balance in hand Oct. 1, 1908 .. .. .. .. .. | 94 | 14 | 5 | 1908. | | | |
| Balance in hand on Account of *Journal* .. | 13 | 18 | 11 | Oct. 11.—Grant to Librarian .. .. .. | 5 | 5 | 0 |
| Members' Subscriptions | 173 | 5 | 0 | Oct. 11.—Stamps .. | 1 | 0 | 0 |
| Interest on Deposit Notes | 3 | 0 | 2 | Dec. 8.—Hall, etc., and Fitzgerald .. .. | 0 | 15 | 9 |
| Excess on a Subscription for *Journal* .. .. | 0 | 5 | 6 | ,, 22—For Lantern .. | 1 | 11 | 6 |
| | | | | 1909. | | | |
| | | | | Jan. 7.—Farr, Stamps, etc. .. .. .. | 2 | 0 | 0 |
| | | | | April 20.—To *Journal* Account .. .. | 0 | 5 | 6 |
| | | | | May 7.—Editorial Sec. (174 members, 1909) | 43 | 10 | 0 |
| | | | | ,, 7.—Editorial Sec. (20 members, 1908) | 5 | 0 | 0 |
| | | | | June 23.—Porter .. | 1 | 3 | 4 |
| | | | | ,, 23.—Reporter .. | 0 | 18 | 0 |
| | | | | Sept. 23.—Rent, Telephone, etc. .. .. | 57 | 6 | 0 |
| | | | | ,, 23.—Library Grant | 65 | 0 | 0 |
| | | | | ,, 10.—Printing .. | 3 | 14 | 6 |
| | | | | ,, 30.—Hon. Sec., Petty Cash .. .. | 1 | 18 | 4 |
| | | | | 1908. | | | |
| | | | | Dec. 30.—To *Journal* .. | 13 | 18 | 11 |
| | | | | 1909. | | | |
| | | | | July 14.—Cheque Book | 0 | 2 | 6 |
| | | | | Oct.—Balance .. .. | 81 | 14 | 8 |
| | £283 | 4 | 0 | | £283 | 4 | 0 |

---

*November 10th.*

Dr. James Swain, President, in the Chair.

Dr. Charles read notes on a case of **Kala Azar,** and on a case of **Myesthenia Gravis,** and showed the patients. Dr. Michell Clarke referred to a case of Kala Azar. He had shown a specimen of the blood from the spleen of the patient to the Society.—Dr. Rendle Short showed specimens of the organism obtained from a case of Oriental Sore, which organism was the same as that found in Kala Azar.

Dr. Walter Swayne read a paper on **The Treatment of Retroversion of the Gravid Uterus.** The affection occurred frequently, but in many cases the malposition became rectified spontaneously, though in others it was troublesome, and might be fatal. If recognised early the difficulties of treatment would be small. It was most troublesome in primiparæ. These patients might attribute the urinary symptoms to the normal state of pregnancy. In treatment the first thing was to evacuate the bladder, which opposed reposition of the uterus. He had seen one case in which the bladder was aspirated supra-pubically because the catheter previously used had been passed into a false passage. The urethra in these cases was stretched, and the catheter might have to be passed several inches to draw off the urine. If attempted reduction with the fingers in the vagina was unsuccessful, the cervix should be seized in vulsellum forceps, and the finger used in the rectum. Thirdly, the uterus might be evacuated, but this procedure was difficult owing to difficulty in dilating the cervix. The alternative was to reduce by laparotomy. This was suggested by Dr. John Williams, of Cardiff, twelve or thirteen years ago. The speaker gave details of three cases illustrating the modes of treatment. He considered that laparotomy was the best treatment if the bladder trouble was severe, and if adhesions were thought to be present. The latter were difficult to diagnose. Incision of the bladder to permit irrigation and escape of sloughs was sometimes indicated.—Dr. ARTHUR FELLS had met with several cases in which he had failed to replace the uterus with the fingers, the patient being in the left lateral position, but had succeeded by placing the patient in the knee-elbow position and making sustained pressure upon the uterus by means of a good sized sponge held in a sponge-holder.—Dr. R. C. LEONARD inquired as to the period of pregnancy of the speaker's cases.—Mr. D. C. RAYNER had performed laparotomy for a case in which he could not otherwise replace the uterus. The impaction had been due to the existence of a fibroid. He had to give up an attempt to enucleate the fibroid owing to the free bleeding, and had to perform hysterectomy instead.—The PRESIDENT asked if it would not be better to perform laparotomy as a routine, rather than attempt to evacuate the uterus.—Dr. SWAYNE, replying, said he considered laparotomy was not dangerous in pregnancy. It was doubtful if the bladder in some cases could be sufficiently well drained without incision. Reduction was impossible without separating adhesions, if such existed. He would prefer to proceed by laparotomy rather than by evacuating the uterus, if the cervix was high up in the later periods of the affection.

Dr. F. H. EDGEWORTH read notes of a case of **Recovery from Tuberculous Meningitis,** and showed the patient. The patient was a woman, 22 years of age, who was admitted to the Bristol Royal Infirmary on October 26th. For fourteen days previously she had had intense headache, preventing sleep. There was marked tenderness over the head, slight fever, and double optic neuritis. No other abnormal physical signs were present. Spinal puncture evacuated a test-tubeful of clear fluid, without cells in it, and without micro-organisms. Steady improvement followed the puncture. He considered that the woman had meningitis. Vaccination of the arm with tuberculin gave a marked positive reaction.—Mr. HARSANT remarked that a recent writer in "Brain" had estimated that there were 1 per cent. of recoveries in tubercular meningitis.—Dr. B. M. H. ROGERS believed recovery was probably commoner than was thought. During last year two cases at the Bristol Children's Hospital had recovered.—Dr. WATERHOUSE asked if thrombosis of cerebral vessels could be excluded in the speaker's case. In adults, apart from phthisis, tubercular meningitis was very rare. He had seen a case recover under treatment with morphia. He asked if syphilis could be excluded.—Dr. J. A. NIXON also raised the question as to exclusion of syphilis. He had seen one case of headache, tenderness to percussion and optic neuritis, which proved to be syphilitic.—Dr. EDGEWORTH replied that there was no evidence of syphilis, and that the patient was not anæmic as in cases of thrombosis, and the optic neuritis of the patient was more intense than in that affection.

Dr. A. RENDLE SHORT read a paper on **Iodoform and Thyroidism.**[1] He showed that in one group of cases of iodoform poisoning the symptoms were the same as those of acute thyroidism, and gave details of a case in which the use of iodoform in the treatment of a carbuncle had been quickly followed by symptoms of exophthalmic goitre.—Dr. SHINGLETON SMITH alluded to the way in which iodoform had been administered continuously for months, in former years, in the treatment of tuberculosis. These cases did not develop the symptoms of thyroidism. He remembered one patient developing weakness in the lower limbs under the treatment.—Dr. CLAYTON remarked upon the interest of the paper. He had been taught that no drug produced fever, but here was one which apparently did so, but only secondarily, by stimulating the activity of the thyroid gland.—Dr. WATERHOUSE asked how boiling the water could prevent it causing goitre, if it caused goitre through its iodine content as suggested.

Dr. WATSON WILLIAMS read notes of a case of **Radical Osteo-Plastic Operation for Double Fronto-Ethmoidal Sinus Suppuration,** and showed the patient. The patient had had headache for ten years. Twelve months previously the antra and sphenoidal sinuses had been opened up; later the anterior ends of the middle turbinates had been removed, and the frontal sinuses then treated by irrigation, the ducts being unusually patent. The speaker described his operative technique. Both frontal

1 We hope to publish the paper in our next issue.

sinuses had been dealt with at one operation. On the right side, following Killian's method, the roof of the orbit had been widely removed. The patient had lost her headache and other symptoms, while scarring was very slight indeed.—Dr. GREEN remarked that he had witnessed the operation, and had been struck with its extent and severity. The scarring was remarkably small.—The PRESIDENT would have thought it risky to expose the orbital tissues in the way described.—Dr. WILLIAMS replied that removing the roof of the orbit was not found to lead to complications. It was very important not to irrigate for several days after the operation.

J. LACY FIRTH.

J. A. NIXON, *Hon. Sec.*

